# Arthrogryposis multiplex congenital (AMC) in a three year old boy: differential diagnosis with distal arthrogryposis: a case report

**DOI:** 10.1186/1757-1626-2-9403

**Published:** 2009-12-30

**Authors:** Zoran S Gucev, Nada Pop-Jordanova, Gordana Dumalovska, Orhideja Stomnaroska, Gorgji Zafirovski, Velibor B Tasic

**Affiliations:** 1 Medical Faculty Skopje, 50 Divizija BB, 1000 Skopje, Former Yugoslav Republic of Macedonia

## Abstract

**Introduction:**

Arthrogryposis multiplex congenital (AMC) is characterized by contractions of multiple joints present at birth. The involved muscles are partially or totally replaced by fat or fibrous tissue. Talipes equinovarus and scoliosis are also frequently reported.

**Case presentation:**

This 2 year was born after uneventful pregnancy, with normal birth weight and length. The parents are unrelated, young and healthy. No malformations or mental retardation have been reported in the family. Since his birth a specific posture was noted: internal rotation at the shoulders, extension at the elbows, and flexion at the wrists. In addition, the child has a severe equinovarus deformity of the feet. Syndactily between II and III finger was also noted. His face is round with a frontal midline capillary hemangioma, while his jaw appears to be small. Mental development is normal. The karyotype is: 46, XY.

**Conclusions:**

About 150 syndromes have arthrogryphosis as a presenting sign. AMC is a distinct entity and distinction with the distal forms of arthrogryphosis can be difficult, since there is a considerable clinical and genetic heterogeneity. A comprehensive musculoskeletal evaluation and genetic consultation is necessary.

## Introduction

Stiff joints present from birth (arthrogryphosis) are a frequent clinical finding. The frequency of the phenomenon stems from the fact that arthrogryphosis can be a syndrome per se, or it can be a part of many other syndromes, malformations or muscular or neurological conditions. Such a vast clinical diversity is accompanied, at least in some of those specific conditions by a remarkable genetic heterogeneity. In some of the characteristic clinical forms the diagnosis is straightforward. In others, finding a precise diagnosis can be a challenge. The different treatments for particular conditions, as well as the possibility for genetic counseling for some of the arthrogryphosis conditions render the very precise diagnosis necessity.

We here present a child with arthrogryphosis congenital multiplex (AMC) and its delineation from the other arthrogryphosis conditions, in particular with the syndromes of the distal arthrogryphosis (DA).

## Case Presentation

This 2 year old boy was diagnosed with AMC at the age of 10 months. He was born after uneventful pregnancy, with normal birth weight and length. The parents are unrelated, young and healthy, and the family does not have a history of malformations or mental retardation. A peculiar body posture is evident: internal rotation at the shoulders, extension at the elbows, and flexion at the wrists. A severe equinovarus deformity of the feet and syndactily between II and III finger are also present. A frontal facial midline capillary hemangioma, and a small jaw are also present. His mental development is normal retarded. Ultrasound of the kidneys is uneventful. Creatinin kinase concentrations in serum were normal. Karyotype is normal male: 46, XY. Radiography revealed scoliosis and talipes equinovarus.

## Discussion

Since the first descriptions [1] AMC was described as limited flexion of joints of the arms and neck and with absent flexion creases of the fingers. In addition, talipes equinovarus and scoliosis and were also reported. In a review article, Hall et al. (1983)[2] found congenital contracture syndrome in 135 of 350 patients. An early observation included a specific positioning present at birth with internal rotation at the shoulders, extension at the elbows, and flexion at the wrists. Severe equinovarus deformity of the feet is frequently present [3]. A frontal midline capillary hemangioma, small jaw, round face and normal intelligence are also found to be typical. Twins have been described, but the condition is mainly sporadic. Our patient has all the features belonging to the AMC. In addition, since no other family member has joint contractures or a malformation our child is a sporadic case of AMC.

Besides the classic AMC, other distinct types of X-linked arthrogryposis have been described. DA1 is a distal form of AMC characterized by autosomal dominant inheritance, intrafamilial variability and involvement primarily of the hands and feet. The position of the hands is specific: the fingers are medially overlapping and ulnarly deviated, the fists are clenched. No associated visceral anomalies have been reported, while the intelligence is normal [3,4].

Distal arthrogryposis type 2B is a clinically and genetically heterogeneous disorder characterized by clenched fist, overlapping fingers, camptodactyly, ulnar deviation, and positional foot deformities from birth. This type of distal arthrogryphosis can be caused by mutations in TNN3 gene which encodes tropinin T and I.

DA2B shares features with DA1 and Freeman-Sheldon syndrome (FSS), also known as DA2A [5-7]. DA2B is characterized by contractures of the fingers, toes, wrist, ankles, knees, and elbows with a lack of interphalangeal creases and bilateral ptosis [3,8]. Later, Bamshad et al. (1996) renamed this form of DA as DA5 [9].

There is a considerable genetic heterogeneity among various forms of DA. Sung et al. (2003) reported that DA1 can be caused by mutation of the TPM2, the gene encoding beta-tropomyosin, while DA2B can be caused by mutations in the TNNI2 gene, which encodes an isoform of troponin I [10,11]. The distal arthrogryposis type 2A can be caused by mutation in the MYH3 gene. Mutations in this gene can also cause distal arthrogryposis type 2B (DA2B), also known as Sheldon-Hall syndrome. An abnormal x-ray appearance of the floor of the anterior cranial fossa of the skull, and facial characteristics withe deep-sunken eyes, hypertelorism, increased philtrum length, small nose and nostrils, and a small mouth are a specific distinction of this DA type [12]. Bamshad et al. (1996) denoted Freeman-Sheldon syndrome as DA2 (later DA2A)[9].

DA2A which includes in its phenotype ptosis, dwarfism, small contracted mouth, and proximal and distal joint contractures was found to have MYH3 gene mutations. On the other hand, Toydemir et al. (2006) found a mutation in the MYH3 gene in 26 of 28 FSS cases [13].

Another form of DA, DA2E, characterized by small mouth and jaw with limited jaw movement, with horizontal depression above the chin, microcephaly and severe flexion contractures of the hands and feet [3,12].

Congenital contractural arachnodactyly (CCA) is a rare, autosomal dominant disorder characterized by contractures, arachnodactyly, scoliosis, and crumpled ears with overlapping features with Marfan syndrome. Wang et al. (1995) identified point mutations in the FBN2 gene in cases of CCA [14]. Bamshad et al. (1996) referred to this disorder as distal arthrogryposis type 9 (DA9)[9].

Arthrogryposis multiplex congenita can also have neurogenic origin. This form of arthrogryphosis is also genetically heterogeneous. It includes a subgroup which is allelic to spinal muscular atrophy type I, or Werdnig-Hoffmann disease (SMA1).

Some AMC forms are mild [15] and limited to the lower extremities, others (DA 10) have only short tendo calcaneus [16,17]. DA5 is arthrogryposis with oculomotor limitation and electroretinal abnormalities [18], DA4 (DAIID) is a form with severe scoliosis [3]. AMC can also be of neurogenic type, resulting from GLE1 gene mutations [19], or can have a specific whistling face (Illum syndrome)[20].

## Conclusions

AMC is a distinct entity that needs to be delineated from the other arthrogryphosis types (~10 types so far) and other syndromes in which stiff joints are a part of the phenotype (~150 syndromes). In particular, the distinction with the distal forms of arthrogryphosis can be challenging. A considerable clinical and genetic heterogeneity is noted in almost all arthrogryphosis types. Therefore, a comprehensive musculoskeletal evaluation and genetic consultation is necessary.

## Consent

Written informed consent was obtained from the father of the patient for publication of this case report.

## Competing interests

The authors declare that they have no competing interests.

## Authors' contributions

All of the authors were involved in the clinico-radiographic assessment and finalizing the paper. All authors have red and approved the final version of the paper.

## References

[B1] Lacassie Y Sack GH Jr McKusick VA An autosomal dominant form of arthrogryposis multiplex congenita (AMC) with unusual dermatoglyphics. (Abstract) Birth Defects Orig Art Ser 1977 XIII 3B 246 247

[B2] Hall JG Reed SD Driscoll EP Part 1. Amyoplasia: a common, sporadic condition with congenital contractures Am J Med Genet 1983 15 571 590 10.1002/ajmg.1320150407 6614047

[B3] Hall JG Reed SD Greene G The distal arthrogryposes: delineation of new entities--review and nosologic discussion Am J Med Genet 1982 11 185 239 10.1002/ajmg.1320110208 7039311

[B4] Daentl DL Berg BO Layzer RB Epstein CJ A new familial arthrogryposis without weakness Neurology 1974 24 55 60 4855665 10.1212/wnl.24.1.55

[B5] Krakowiak PA O'Quinn JR Bohnsack JF Watkins WS Carey JC Jorde LB Bamshad M A variant of Freeman-Sheldon syndrome maps to 11p15.5-pter Am J Hum Genet 1997 60 426 432 9012416 PMC1712403

[B6] Shrimpton AE Hoo JJ A TNNI2 mutation in a family with distal arthrogryposis type 2B Europ J Med Genet 2006 49 201 206 10.1016/j.ejmg.2005.06.003 16497570

[B7] Kimber E Tajsharghi H Kroksmark A-K Oldfors A Tulinius M A mutation in the fast skeletal muscle troponin I gene causes myopathy and distal arthrogryposis Neurology 2006 67 597 601 10.1212/01.wnl.0000230168.05328.f4 16924011

[B8] Friedman BD Heidenreich RA Distal arthrogryposis type IIB: further clinical delineation and 54-year follow-up of an index case Am J Med Genet 1995 58 125 127 10.1002/ajmg.1320580207 8533802

[B9] Bamshad M Jorde LB Carey JC A revised and extended classification of the distal arthrogryposes Am J Med Genet 1996 65 277 281 10.1002/(SICI)1096-8628(19961111)65:4<277::AID-AJMG6>3.0.CO;2-M 8923935

[B10] Sung SS Brassington A-ME Grannatt K Rutherford A Whitby FG Krakowiak PA Jorde LB Carey JC Bamshad M Mutations in genes encoding fast-twitch contractile proteins cause distal arthrogryposis syndromes Am J Hum Genet 2003 72 681 690 10.1086/368294 12592607 PMC1180243

[B11] Sung SS Brassington A-ME Krakowiak PA Carey JC Jorde LB Bamshad M Mutations in TNNT3 cause multiple congenital contractures: a second locus for distal arthrogryposis type 2B. (Letter) Am J Hum Genet 2003 73 212 214 10.1086/376418 12865991 PMC1180583

[B12] Hall JG Truog WE Plowma DL A new arthrogryposis syndrome with facial and limb anomalies Am J Dis Child 1975 129 120 122 1130329 10.1001/archpedi.1975.02120380090021

[B13] Toydemir RM Rutherford A Whitby FG Jorde LB Carey JC Bamshad MJ Mutations in embryonic myosin heavy chain (MYH3) cause Freeman-Sheldon syndrome and Sheldon-Hall syndrome Nature Genet 2006 38 561 565 10.1038/ng1775 16642020

[B14] Wang M Tsipouras P Godfrey M Fibrillin-2 (FBN2) mutation in congenital contractural arachnodactyly. (Abstract) Am J Hum Genet 1995 57 A231

[B15] Zori RT Gardner JL Zhang J Mullan MJ Shah R Osborn AR Houlden H Wallace MR Roberts S Yang TP Newly described form of X-linked arthrogryposis maps to the long arm of the human X chromosome Am J Med Genet 1998 78 450 454 10.1002/(SICI)1096-8628(19980806)78:5<450::AID-AJMG10>3.0.CO;2-E 9714012

[B16] Levine MS Congenital short tendo calcaneus Am J Dis Child 1973 125 858 859 4708278 10.1001/archpedi.1973.04160060062014

[B17] Stevenson DA Swoboda KJ Sanders RK Bamshad M A new distal arthrogryposis syndrome characterized by plantar flexion contractures Am J Med Genet 2006 140A 2797 2801 10.1002/ajmg.a.31528 PMC3244115 17103435

[B18] Beals RK Weleber RG Distal arthrogryposis 5: a dominant syndrome of peripheral contractures and ophthalmoplegia Am J Med Genet 2004 131 67 70 10.1002/ajmg.a.30289 15389706

[B19] Nousiainen HO Kestila M Pakkasjarvi N Honkala H Kuure S Tallila J Vuopala K Ignatius J Herva R Peltonen L Mutations in mRNA export mediator GLE1 result in a fetal motoneuron disease Nature Genet 2008 40 155 157 10.1038/ng.2007.65 18204449 PMC2684619

[B20] Illum N Reske-Nielsen E Skovby F Askjaer SA Bernsen A Lethal autosomal recessive arthrogryposis multiplex congenita with whistling face and calcifications of the nervous system Neuropediatrics 1988 19 186 192 10.1055/s-2008-1052443 3205375

